# A Sensitive Period for Automaticity Rather than Ability: Automatic Visuospatial Coding of Touch Requires Visual Experience in Early Life

**DOI:** 10.1111/desc.70300

**Published:** 2026-11-03

**Authors:** Stephanie Badde, Suddha Sourav, Kabilan Pitchaimuthu, Bhavana Kolli, Waqar Khan, Ramesh Kekunnaya, Brigitte Röder

**Affiliations:** ^1^ Biological Psychology and Neuropsychology University of Hamburg Hamburg Germany; ^2^ Department of Psychology Tufts University Medford Massachusetts USA; ^3^ Department of Medicine and Optometry Linnaeus University, Sweden; ^4^ Child Sight Institute, Jasti V Ramanamma Children's Eye Care Center LV Prasad Eye Institute Hyderabad India

## Abstract

Early visual experience can profoundly shape tactile perception. Sighted and late blind but not congenitally blind humans automatically link touch to a visuospatial reference frame—evidenced by a tendency to assign tactile stimuli to the wrong hand when the hands are crossed. Here, we tested for a sensitive period during which visual experience irrevocably shapes the automaticity of visuospatial coding of touch. To this aim, tactile spatiotemporal perception was measured in individuals who had been temporarily blind due to bilateral dense cataracts that were either congenital or developed in childhood. Participants reported the location of the first of two successive tactile stimuli—one on each hand—resting their arms in a crossed or uncrossed position. In Experiment 1, only tactile stimulus pairs were presented (unimodal context). In Experiment 2, pairs of tactile stimuli were interleaved with tactile‐auditory and auditory pairs (crossmodal context). In the unimodal context, individuals with restored congenital cataracts—reminiscent of those with congenital blindness—did not exhibit the typical impairment with crossed hands, confirming irrevocable effects of early vision on tactile perception. In stark contrast, in the crossmodal context, hand crossing markedly impaired their tactile judgments. Here, individuals with restored congenital cataracts performed similarly to typically‐sighted individuals. Thus, the genuine ability to code touch visuospatially did not depend on early vision. Participants with restored childhood‐onset cataracts and typically‐sighted participants showed comparable crossing effects in both contexts. In sum, the present study reveals that the automaticity rather than the ability to code touch visuospatially requires early visual experience.

## 1 Introduction

Sensory experience in one modality can shape the neural circuits that underlie perception in other modalities (Bavelier and Neville 2002; Knudsen and Brainard 1991). This influence is especially strong during sensitive periods of development; functional development remains incomplete if typical sensory input has been missing during these epochs of life (De Villers‐Sidani and Merzenich 2011; Knudsen 2004).

Visual experience strongly impacts tactile spatiotemporal perception: Typically‐sighted humans automatically link touch to a visuospatial reference frame–evidenced by a tendency to assign tactile stimuli to the wrong hand when the hands are crossed (Badde et al. 2019; Heed and Azañón 2014; Shore et al. 2002; Yamamoto and Kitazawa 2001). However, congenitally blind individuals did not exhibit the same impairment when localizing the first of two tactile stimuli on crossed hands (Crollen et al. 2017; Crollen, Lazzouni, et al. 2017; Röder et al. 2004). Furthermore, infants born with severe visual impairments did not exhibit the same impairments as sighted infants when orienting toward a tactile stimulus on crossed hands (Gori et al. 2021). This link between touch and visual space appears to depend on visual experience during a sensitive period in early life: Late blind participants who lost their vision after childhood showed hand‐crossing effects indistinguishable from those of typically‐sighted participants (Röder et al. 2004).

To provide evidence for sensitive periods in brain development, it is necessary to demonstrate that restoring typical sensory experience does not reinstate typical sensory functions (Knudsen 2004). In humans, investigating individuals with a transient phase of congenital blindness during infancy or childhood provides a unique key to uncover sensitive periods in human brain development (Maurer et al. 2007a; Röder et al. 2021).

Consistent with a sensitive period, two independently tested participants whose sight was restored after two (Ley et al. 2013) and seven years (Azañón et al. 2018) of temporary congenital blindness due to bilateral dense cataracts showed no effects of hand position on performance in the same tactile spatiotemporal order judgment task that had been used in congenitally blind individuals (Röder et al. 2004). Despite years of visual experience after their cataract surgery, they performed like congenitally blind individuals (Azañón et al. 2018; Ley et al. 2013). In contrast, children born with bilateral cataracts whose sight was restored within the first five months of life exhibited crossing effects indistinguishable from those of typically sighted individuals, suggesting that this sensitive period might end after the first years of life (Azañón et al. 2018). In sum, the emerging pattern of results suggests a sensitive period—possibly before the age of 2 years—during which visual experience irrevocably shapes tactile spatial perception.

An intuitive interpretation of the lack of body posture effects on tactile perception in congenitally blind individuals is that early vision is required to consolidate the neural circuits that relate the body to external space. Yet, congenitally blind humans are able to localize tactile and auditory objects in external space. For example, they successfully localized their crossed hands in reference to sounds (Röder et al. 2007). Moreover, congenitally blind humans were able to code touch in visuospatial coordinates when the task required (Schubert et al. 2017) or stressed (Heed et al. 2015) these. Thus, we here hypothesized that the automaticity, rather than the ability to visuospatially code touch, depends on early visual input.

Similar, seemingly contradictory findings as for congenitally blind humans have emerged with respect to children's ability to code touch visuospatially. Infants and children were able to orient to and act upon tactile stimuli on their hands independently of hand position (Bremner et al. 2008). Yet, when tested in the same tactile spatiotemporal order judgment task that revealed differences in tactile spatial coding in congenitally blind adults, children younger than six years did not show crossing effects, i.e., no evidence of visuospatial coding of touch (Pagel et al. 2009). However, when the task was altered to emphasize locations in external space, children of the same age relied on visuospatial coding of touch (Begum Ali et al. 2014). Thus, young children might not automatically link touch to external space, but be able to do so. Consequently, the automatic use of visuospatial codes in tactile processing rather than the genuine ability to link touch to a visuospatial reference frame might develop during a sensitive period in childhood (Röder et al. 2014).

Support for the idea of a sensitive period for the automaticity rather than the ability of visuospatial coding of touch comes from the performance of an individual who underwent treatment for congenital cataracts at the age of two years. As mentioned above, the participant did not show a crossing effect in a tactile spatiotemporal order judgment task. However, hand crossing affected tactile perception in a crossmodal context (Ley et al. 2013): With crossed hands, this participant's performance in a tactile spatial discrimination task was more impaired by visual distractors in the same hemifield, that is, in the same visuospatial region, than by those in the other hemifield. Thus, this sight‐restored participant did not code touch in a visuospatial reference frame in a tactile task but relied on such a reference frame for touch localization in a crossmodal context, albeit in a different task.

Here, we tested the emerging hypothesis that a delayed onset of visual experience prevents the development of an automatically activated link of touch to a visuospatial reference frame in humans. To contrast the ability to represent touch in a visuospatial reference frame against the automaticity of doing so, participants completed the same tactile spatiotemporal order judgment task used in previous studies in two sensory contexts: In Experiment 1, only tactile stimulus pairs were presented (unimodal context). Thus, the task provided no incentive to link touch to external space; hence, the use of visuospatial coordinates—indicated by impaired performance with crossed hands—would suggest the automaticity of such a link. In Experiment 2, tactile stimulus pairs were presented interleaved with auditory and tactile‐auditory stimulus pairs (crossmodal context). Therefore, two‐thirds of stimulus pairs required stimulus localization in the external world. Comparing crossing effects for tactile stimulus pairs in Experiment 1 and Experiment 2 allowed us to assess a possible context‐dependent use of visuospatial coding of touch. If the ability to code touch in visuospatial coordinates develops conditional on visual experience during infancy, the performance of sight‐restored participants with a history of congenital cataracts should not be affected by hand position in both experiments. However, if the automaticity of visuospatial coding depends on early vision, they might show crossing effects in the cross‐modal context of Experiment 2. To control for the potential effects of a history of cataracts on performance, sight‐restored individuals with cataracts that had developed in childhood were tested in addition to age‐matched typically sighted participants.

## 2 Methods

### 2.1 Participants

Nineteen individuals with a history of bilateral dense congenital cataracts participated in the study. Seven of them participated in both experiments, five took part only in Experiment 1, and the remaining seven participated solely in Experiment 2. Thirteen individuals with treated developmental cataracts (childhood onset cataracts) were recruited as controls. Six of them completed both experiments, four took part only in Experiment 1, and three participated solely in Experiment 2. Twenty‐nine participants with a history of cataracts were recruited and tested at the LV Prasad Eye Institute in Hyderabad, India. Three sight‐restored participants with a history of congenital cataracts were recruited and tested at the University of Hamburg, Germany. Congenital cataracts were treated between two and 252 months after birth (*M* = 57 months; for further information about the sample, see Table S1). The diagnosis of congenital cataracts was confirmed by an optometrist and/or ophthalmologist based on the medical records. Since cataracts were sometimes diagnosed years after birth, additional criteria such as the presence of nystagmus, strabismus, the density of the lenticular opacity, lack of fundus visibility prior to surgery, a family history of congenital cataracts, and parents’ reports were used to confirm the cataract etiology.

The performance of participants with restored sight was compared to that of typically sighted participants. The distributions of age, sex, and handedness were similar between sight‐restored and typically‐sighted groups (see Table S1). Adult and adolescent typically‐sighted participants were recruited and tested in the same location as the sight‐restored participants. Typically‐sighted participants younger than twelve years were recruited in Hamburg, resulting in a slightly uneven distribution of locations across groups. Using an alternative composition of the typically‐sighted groups that prioritizes location over age did not alter the pattern of the results.

Participants or their legal guardians were reimbursed for travel expenses and other costs associated with taking part in the study. Minors received a small present. The study was approved by the the ethics board of the German Psychological Society (Ref. BR 07‐2011) and the ethics board of the LV Prasad Eye Institute (Ref. LEC 11‐086). Before beginning the experiment, all participants or, if applicable, their legal guardians gave written informed consent.

### 2.2 Setup and Stimuli

Participants were seated at a table, with their arms resting either crossed or uncrossed on the surface (Figure 1A). They roughly aligned each hand with a speaker (Companion 2 Series III, Bose, Framingham, MA, USA) positioned 14 degrees (15 cm at 60 cm distance) to the left and right of their body midline. A custom‐made, noise‐attenuated tactile stimulator was affixed to the fingertips of both index fingers using medical tape. Tactile stimuli were 15 ms‐long vibrations with a frequency of 100 Hz; auditory stimuli were 15 ms‐long white noise bursts. The loudness of the auditory stimuli was adjusted to roughly match the intensity of the tactile stimuli and kept constant throughout the study. All stimuli were clearly above their respective detection thresholds. To mask any residual noise produced by the tactile stimulators, constant white noise was played from two additional centrally located speakers, and participants’ hands were placed inside oven mitts.

**FIGURE 1:**
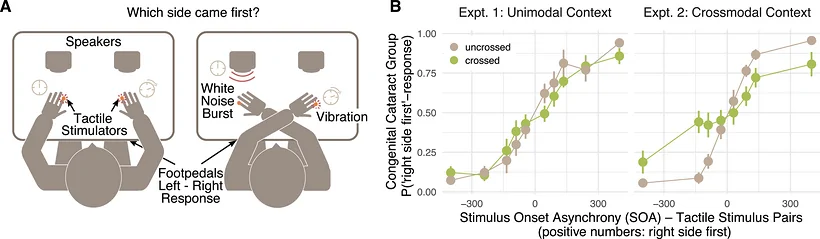
Experimental setup and performance of individuals with a history of congenital cataracts in spatiotemporal order judgments of tactile stimulus pairs in Experiments 1 and 2. (A) Participants positioned their hands either crossed or uncrossed and roughly aligned with two speakers. In each trial, two stimuli were presented—one after the other, one on either side. Participants indicated the location of the first stimulus by activating the spatially corresponding foot pedal. In Experiment 1, only tactile stimulus pairs were presented; in Experiment 2, tactile stimulus pairs were interleaved with tactile‐auditory and auditory stimulus pairs (results for the latter two are shown in Figure 3). (B) Performance of sight‐restored individuals with a history of congenital cataracts when judging the temporal of order of tactile stimulus pairs in Experiment 1 (left panel) and in Experiment 2 (right panel). Group mean proportions of "right side first"‐responses are shown as a function of the tactile stimulus onset asynchrony (SOA; positive values indicate "right side first" stimulus pairs) separately for uncrossed (beige) and crossed (green) hand positions. Errors bars indicate standard errors of the mean.

A fixation cross was displayed on a monitor positioned centrally in front of the participants. Throughout the experiment, participants positioned both feet on custom‐built foot pedals. Four sight‐restored participants struggled to control the foot pedals. These participants and typically‐sighted participants matched to these four responded by raising one hand. The hands and the buttons were marked with colored stickers to facilitate the experimenter's data entry.

The experiment was controlled by a custom‐written script for Presentation (Version 17.1.05, Neurobehavioral Systems, Inc., Berkeley, CA, USA). The experimental software recorded responses and interfaced with custom‐built hardware to drive the stimulators via a multichannel digital input‐output device (NI‐DAQ 6509, National Instruments, Austin, TX, USA).

### 2.3 Procedure

Two stimuli, one on each side, were presented successively in each trial. Participants indicated the location of the first stimulus by lifting their heel off the spatially corresponding foot pedal. Responses had to be withheld until the second stimulus appeared. Response times were capped at three seconds; trials without a valid response within this timeframe were repeated at the end of the block (1% of trials), unbeknownst to the participants. The next trial started two seconds after the response was recorded.

In addition to written instructions, participants received oral instructions in their primary language before beginning the experiment. They completed ten practice trials at the beginning of the experiment and again when they first changed their arm position. During these trials, stimuli were presented with an offset of 800 ms. If participants felt uncertain about the task or made multiple mistakes, the practice trials were repeated until they felt confident enough to continue. Aside from the practice trials, participants did not receive any feedback.

In Experiment 1, solely tactile stimulus pairs were presented. The location of the first stimulus (left and right) and the stimulus onset asynchrony (SOA; 45, 90, 135, 240, 400, 800, and 1500 ms) varied randomly across trials. After each block, participants changed the position of their hands from uncrossed to crossed or vice versa; the initial position was counterbalanced across participants. Each of the 28 stimulus conditions (2 locations of the first stimulus x 7 SOAs x 2 hand positions) was repeated 12 times. The experiment was divided into eight blocks and lasted approximately 20–30 min.

In Experiment 2, tactile and auditory stimuli were presented. The modality of the two stimuli per trial (tactile‐tactile, tactile‐auditory, auditory‐tactile, and auditory‐auditory), the location of the first stimulus (left and right), and the SOA (45, 90, 135, and 240 ms) varied randomly across trials. Hand position (uncrossed and crossed) was maintained for three consecutive blocks; the initial position was counterbalanced across participants. Each of the 64 stimulus conditions (4 modality combinations x 2 locations of the first stimulus x 4 SOAs x 2 hand positions) was repeated 10 times. The experiment was split into 20 blocks of 32 trials each and took about 60 min to complete.

Sight‐restored participants who completed both experiments first took part in Experiment 1 and completed Experiment 2 during a later session. Typically‐sighted participants who completed both experiments did so in random order.

### 2.4 Data Analysis

Responses in unimodal tactile and auditory trials were recoded into “right side first” responses: “1” if the participant indicated that the stimulus on the right side had been presented first, and “0” otherwise. SOA was recoded so that negative values indicate the first stimulus was on the left side, while positive values indicate it was on the right side. Response probabilities naturally varied with SOA, in addition to our variables of interest. To reduce the number of predictors in the statistical model by more than half, we quantified participants’ sensitivity to the spatiotemporal order of the stimuli, that is, the relationship between SOA and performance, in a single variable. To this aim, we conducted a probit analysis (Finney 1947), that is, we conducted a linear regression predicting the probit‐transformed proportion of “right side first” responses for each SOA. The analysis was limited to SOAs for which performance had not reached an asymptote on a group level, ensuring that the estimated probit regression coefficients remained unaffected by participants’ lapse rates.

Responses in tactile‐auditory trials were recoded into “auditory stimulus first” responses: “1” if participants indicated that the auditory stimulus was presented first, and “0” if their response indicated they perceived the tactile stimulus first. The SOAs of these stimuli were recoded so that positive values indicated the auditory stimulus was presented first, while negative values indicated that the tactile stimulus was presented first. A probit regression analysis was conducted on these data to quantify participants’ sensitivity to temporal offsets across spatially separated modalities, as measured by the probit regression slope.

The main analysis compared tactile crossing effects across age‐matched groups and sensory contexts (Experiment 1 vs. Experiment 2). We estimated two linear mixed models of the probit regression slopes with hand position (uncrossed vs. crossed), experiment (Experiment 1 – unimodal context vs. Experiment 2 – cross modal context), and group (Model A: congenital cataract vs. age‐matched typically sighted and Model B: developmental cataract vs. age‐matched typically sighted) as fixed effects and random intercepts. We did not include random slopes in the statistical model, because the modeled data consists of one probit slope per participant and condition. This is insufficient to estimate the 4×4 variance‐covariance matrix of a GLMM with a full random‐effects structure, or the 3×3 or 2×2 matrices of models with reduced structures, i.e., models with random slopes for one or two of the three predictors. Significant interaction effects, which were observed exclusively in the regression model comparing the performance of sight‐restored participants treated for congenital cataracts to that of age‐matched typically‐sighted control participants, were analyzed further using custom contrasts. We first tested for interactions between group and hand position in each experiment, and then we examined hand position effects within each group and experiment.

The first control analysis compared crossing effects in tactile‐auditory trials of Experiment 2 across matched groups. We fitted linear mixed models of the probit regression slopes with hand position and group as fixed effects. The second control analysis examined crossing and group effects in auditory trials of Experiment 2. We fitted linear mixed models of the probit regression slopes with hand position and group as fixed effects.

All analyses were coded in R. Analysis scripts and the de‐identified data are publicly available (https://www.fdr.uni‐hamburg.de/record/18139).

## 3 Results

Participants with a history of congenital cataracts did not show the typical effect of hand position on tactile spatiotemporal order judgments in the unimodal context of Experiment 1 (Figure 1B, left panel). In stark contrast, these participants did exhibit a pronounced performance decline with hand crossing when judging the same tactile stimulus pairs in the crossmodal context of Experiment 2. (Figure 1B, right panel). Conversely, individuals with a history of developmental cataracts and typically sighted participants exhibited performance impairments with crossed compared to uncrossed hands in both unimodal and crossmodal contexts, that is, in Experiment 1 and Experiment 2 (Figure 2).

**FIGURE 2:**
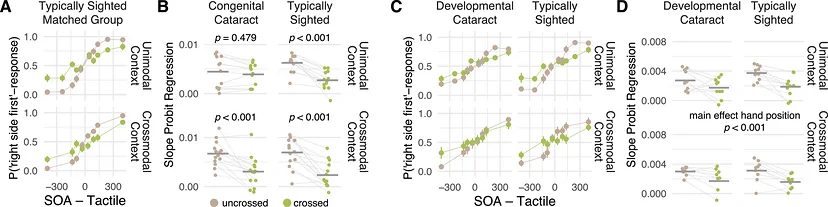
Performance of developmental cataract and typically sighted groups for tactile spatiotemporal order judgments in Experiments 1 and 2. (A) Performance of a group of typically sighted participants matched in age to the group treeated for congenital cataracts in spatiotemporal order judgments of tactile stimulus pairs in Experiment (top panel) and Experiment 2 (bottom panel). (B) A probit regression was performed on the data shown in (A). Individual participant's probit regression slopes are shown separately for uncrossed (beige) and crossed (green) hand positions. Slopes of the congenital cataracts group and the age‐matched typically sighted group from Experiment 1 (top panel) and Experiment 2 (bottom panel) are shown. Grey horizontal lines indicate group mean values; thin grey lines connect individual participants' values across conditions. Post‐hoc contrasts confirmed significant crossing effects for both groups in Experiment 2 but only for the typically‐sighted control group in Experiment 1. (C) Temporal order judgment performance of two additional control groups, individuals who had been treated for cataracts that had developed during childhood, and a group of typically‐sighted individuals age‐matched to the developmental cataracts group. (D) Probit regression slopes corresponding to the data shown in (C). Statistical analysis indicated a significant crossing effect which was not significantly modulated by group or experiment. Post‐hoc checks confirmed significant crossing effects for both groups in each experiment.

**FIGURE 3:**
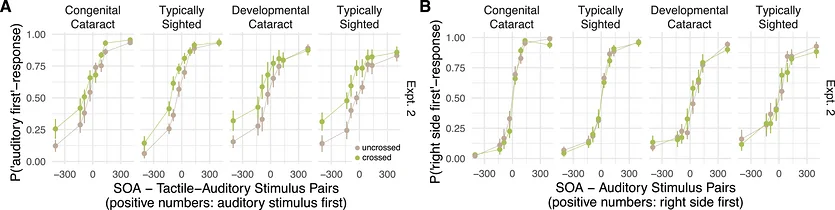
Results for spatiotemporal order judgments of tactile‐auditory and auditory stimulus pairs in experiment 2. (A) Performance of all four groups (participants with a history of congenital cataracts, typically‐sighted participants age‐matched to this group, participants with treated developmental cataracts, and typically sighted participants age‐matched to that group) in trials in which tactile‐auditory stimulus pairs were presented. Responses and the SOA were recoded to indicate the temporal order of the two spatially separated modalities. (B) Performance in trials in which auditory stimulus pairs were presented. All conventions are identical to those in Figures 1 and 2.

Consistently, the first statistical analysis of tactile spatiotemporal order judgments in both experiments confirmed a significant effect of hand position (uncrossed vs. crossed; χ^2^ (1) =  75.11, *p* < 0.001), which was modulated by group (history of congenital cataracts vs. age‐matched typically sighted; χ^2^ (1) =  6.74, *p*  =  0.009) and experiment (Experiment 1 vs. Experiment 2; χ^2^ (1) =  8.08, *p*  =  0.004). Interaction contrasts indicated a significant difference in the crossing effect between groups in Experiment 1 (*t* (54.3) =  2.79, *p*  =  0.007) but not in Experiment 2 (*t* (54.3) =  0.95, *p*  =  0.346). Indeed, whereas for the matched, typically‐sighted group, significant crossing effects were statistically confirmed for both experiments (Experiment 1: *t* (54.3) =  4.66, *p* < 0.001, Experiment 2: *t* (54.3) =  6.54, *p* < 0.001), a significant crossing effect for the group treated for congenital cataracts emerged only in Experiment 2 (Experiment 1: *t* (54.3) =  0.71, *p*  =  0.479, Experiment 2: *t* (54.3) =  5.19, *p* < 0.001). Conversely, the statistical analysis comparing the performance of the group with a history of developmental cataracts against that of their matched typically‐sighted participants confirmed solely a significant main effect of hand position (χ^2^ (1) =  75.11, *p* < 0.001; see Tables S2 and S3 for detailed statistical results).

Consistent with the presence of tactile crossing effects in all groups in Experiment 2, the control statistical analysis of performance in tactile‐auditory trials in Experiment 2 confirmed significant effects of hand position (history of congenital cataracts and age‐matched typically sighted groups: uncrossed vs. crossed; χ^2^ (1) =  16.65, *p* < 0.001; history of developmental cataracts and age‐matched typically sighted groups: uncrossed vs. crossed; χ^2^ (1) =  15.14, *p* < 0.001) but did not indicate significant modulations of the crossing effect across groups (Figure 3A). Finally, the second control analysis of performance in auditory trials of Experiment 2 indicated no significant effects of hand position or group (see Tables S4–S7 for detailed results; Figure 3B), consistent with the absence of general effects of hand crossing on performance and consistent with the absence of general performance differences between age‐matched groups.

## 4 Discussion

Here, we tested the role of visual experience for the ability and automaticity of linking touch to a visuospatial reference frame—quantified through the tendency to misjudge the spatiotemporal order of tactile stimuli on crossed hands. Participants with restored sight who had experienced congenital, sometimes extended periods of pattern blindness due to bilateral, dense cataracts judged the spatiotemporal order of two tactile stimuli on either crossed or uncrossed hands. They, as well as control groups—individuals with a history of developmental cataracts and typically sighted age‐matched participants—completed the task in two different sensory contexts: In Experiment 1, only tactile stimulus pairs were presented, whereas in Experiment 2, tactile stimulus pairs were interleaved with auditory and tactile‐auditory stimulus pairs. Individuals with a history of congenital cataracts did not exhibit an effect of hand position on the spatiotemporal perception of tactile stimuli in the unimodal tactile context (Experiment 1). In stark contrast, in the crossmodal context of Experiment 2, they performed significantly worse with crossed than uncrossed hands, when judging tactile stimulus pairs. Conversely, participants in the different control groups showed impaired performance with hand crossing in either context, suggesting that these participants automatically linked touch to a visuospatial reference frame. In sum, only individuals who had been without vision for a transient phase after birth switched between reference frames depending on the sensory context. This capacity to not activate a visuospatial reference frame of individuals with a history of congenital cataracts suggests that not the ability to use visuospatial coding of touch but rather the automaticity of doing so develops based on early visual experience during a sensitive period.

### 4.1 Sensitive Periods and Sleeper Effects

Many of the present participants with a history of congenital cataracts had their sight restored in early childhood (Table S1). Thus, for them, the onset of vision was delayed but nevertheless occurred during a period of development when typically sighted children do not yet automatically link touch to visual space (Pagel et al. 2009). Such effects of visual deprivation on functions with a later developmental emergence have been termed sleeper effects (Maurer et al. 2007b) and observed for several visual functions, such as visual acuity (Maurer et al. 2007b) and face processing (Röder and Kekunnaya 2021). The common explanation for sleeper effects is that the absence of visual experience in early life prevents the development of neural structures necessary for the function‐specific neural circuit to develop later on.

Sleeper effects reveal a tension between typical development and sensitive periods—time spans in brain development during which the effects of sensory experience are particularly strong, and after which restoring typical sensory functions does not lead to full revision of the effects of atypical sensory experience (Knudsen 2004). On the one hand, prospective developmental studies can provide important insights into the role of sensory information at varying time points during typical development. For example, developmental studies in newborns (Sann and Streri 2007), and children (Gori et al. 2008, Gori et al. 2012) have suggested that for some object features, haptics educates vision, while for others vision educates haptics. However, developmental studies in typically sighted children are not informative about the existence of sensitive periods, that is, the necessity of sensory information during a certain period in human sensory development. On the other hand, sleeper effects demonstrate that the effects of transient periods of atypical sensory experience do not allow us to trace the typical development of perceptual and cognitive functions.

In the present study, the absence of crossing effects in the unimodal context of Experiment 1 did not depend on the age at which the congenital cataracts were treated (Figure S1). Thus, even participants whose congenital cataracts were removed in early childhood—when the automatic use of visuospatial coding for touch is still absent in typical development—did not acquire an automatic link of touch to visuospatial reference frames. These results indicate a sensitive period in early life, in which visual experience initiates changes in the neural circuits that enable the development of an automatic link of touch to a visuospatial reference frame in childhood.

### 4.2 Incomplete Remodeling – A Potential Cause of Increased Flexibility

Individuals who had been congenitally blind due to cataracts were able to toggle between the typical visuospatial coding of touch and a probably body‐based code typically used by congenitally blind adults (Röder et al. 2004). One potential mechanism behind this flexibility could be the absence of the neuronal remodeling that would occur during typical development. Studies in owls have demonstrated that prism‐wearing caused structural changes in the connectivity between the central and external nucleus of the inferior colliculus in juvenile but not adult animals (DeBello et al. 2001). Owls with transient juvenile prism experience had access to a larger number and additional connections in the midbrain due to reduced pruning and the growth of new connections, respectively (DeBello et al. 2001). As adults, they were able to toggle between the atypical and typical mapping between vision and audition, depending on whether or not they were wearing the prims again. Similarly, the reduced or aberrant neuronal remodeling during development might enable individuals previously treated for congenital cataracts to toggle between body‐based coding of touch—as present in congenitally blind individuals—and the typical link of touch to a visuospatial reference frame. Conversely, typically sighted controls and individuals with a history of developmental cataracts might have pruned away or not grew the connections that allow for such a toggling, potentially to the advantage of action control.

Structural brain imaging studies in humans have supported the role of visual experience for structural visual cortex development at circumscribed early stages of human development. Sight‐restored individuals treated for congenital cataracts have thicker visual cortices than typically sighted humans—similar to congenitally blind humans (Hölig et al. 2023; Jiang et al. 2009; Park et al. 2009)—a difference attributed to a lack of pruning or myelination.

### 4.3 Visuospatial Coding of Touch

The present results provide further insights into the mechanisms that lead to errors when localizing touch on crossed hands (or feet). Recent research suggests that these errors are misattributions of touch to the wrong limb (Maij et al. 2020), that is, to a limb placed in the space typically occupied by the touched limb (Badde et al. 2019). A touch on the right hand seems to activate the right hemispace—irrespective of the hand's location in space. This activation was evident in improved visual perception in this space (Azañón and Soto‐Faraco 2008), as well as the misattribution of touch to the wrong limb (Badde et al. 2019) and even the transfer of tactile adaptation (Badde and Heed 2023) to another limb placed in a tactually stimulated limb's typical hemispace.

Findings that congenitally blind humans did not attribute touch to the wrong hand when these are crossed (Crollen, Lazzouni, et al. 2017; Röder et al. 2004) have raised the question of whether this mechanism's development is triggered by visual experience. The emergence of tactile crossing effects in participants with treated congenital cataracts in the cross‐modal context indicates that the neuronal connections underlying the link between somatosensory stimulation and activity in visuospatially organized brain areas develop independently of early visual experience. Rather, early vision seems to shape the multisensory localization network towards to the automatic activation of the connections that implement visuospatial coding of touch, suggesting this leads to an increase in overall localization efficiency despite occasional errors.

### 4.4 General Performance Differences Across Groups

The results figures show a persistent performance difference between the groups with a history of congenital and developmental cataracts. We did not explore this difference because both groups differed in age, and age has been shown to profoundly influence performance in our tasks (Pagel et al. 2009). Consistently, performance of neither of the sight‐restored groups was significantly different from that of age‐matched typically sighted control participants in tactile, tactile‐auditory, and auditory tasks.

## 5 Conclusion

The present study reveals that the automatic link of touch to a visuospatial reference frame that typically emerges in childhood depends on visual experience early after birth.

## Author Contributions

**Stephanie Badde**: conceptualization, methodology, data curation, visualization, writing – original draft, writing – review and editing, software, formal analysis. **Suddha Sourav**: data curation, writing – review and editing, project administration. **Kabilan Pitchaimuthu**: writing – review and editing, project administration, data curation. **Bhavana Kolli**: investigation. **Waqar Khan**: investigation. **Ramesh Kekunnaya**: writing – review and editing, resources. **Brigitte Röder**: conceptualization, methodology, investigation, supervision, funding acquisition, resources, writing – original draft, writing – review and editing.

## Funding

This work was supported by a European Research Council grant to Brigitte Röder (ERC‐2009‐AdG 249425) and by Deutsche Forschungsgemeinschaft grants to Brigitte Röder (DFG Ro 2625/10‐1) and Stephanie Badde (DFG BA 5600/1‐1).

## Conflicts of Interest

The authors declare that no competing interests exist.

## Ethics Statement

The study was approved by the ethics board of the German Psychological Society (Ref. BR 07_2011) and the ethics board of the LV Prasad Eye Institute (Ref. LEC 11‐086).

## Supporting information




**Supporting information**: desc70300‐supp‐0001‐SuppMat.pdf

## Data Availability

Analysis scripts and the de‐identified data are publicly available at https://www.fdr.uni‐hamburg.de/record/18139.
