# Supplementary material for: A Sensitive Period for Automaticity Rather than Ability: Automatic Visuospatial Coding of Touch Requires Visual Experience in Early Life
Source: Dev Sci. 2026 Nov 3;30(1):e70300. doi: 10.1111/desc.70300 (PMC13731914; doi:10.1111/desc.70300)
Supplement: Supplementary file 1 [file DESC-30-e70300-s001.pdf]

Supplementary Material

Table S1. Sample Characteristics

Sight-Restored Participants						Matched Typically-Sighted Participants		
Group-ID	Age at Testing (years)	Age at Surgery (months)	Visual Acuity	Country	Experiment	ID	Age at Testing (years)	Country
CC-A	33	24	20/40	India	Expt. 2	AU	33	India
CC-A	33	24	20/40	India	Expt. 1	AA	35	India
CC-B	26	5	20/126	India	Expt. 2	AO	25	India
CC-C	12	5	20/60	India	Expt. 1	AP	12	India
CC-D	9	2	20/40	India	Expt. 1	AD	9	Germany
CC-E	16	16	20/200	India	Expt. 1	AK	15	India
CC-F	21	252	20/252	India	Expt. 1	AT	23	India
CC-G	30	20	20/400	India	Expt. 2	AM	30	India
CC-H	20	93	CF 1 m	India	Expt. 2	BB	20	India
CC-I	18	222	20/240	India	Expt. 2	AX	19	India
CC-J	13	63	20/120	India	Expt. 2	AK	15	India
CC-K	28	168	20/200	India	Expt. 2	AN	29	India
CC-K	28	168	20/200	India	Expt. 1	AM	30	India
CC-L	12	16	20/40	India	Expt. 2	AP	12	India
CC-L	12	16	20/40	India	Expt. 1	AW	12	India
CC-M	10	12	20/100	India	Expt. 2	AI	10	Germany
CC-N	10	44	20/50	India	Expt. 1	AE	10	Germany
CC-O	18	25	20/60	India	Expt. 2	AR	18	India
CC-O	18	25	20/60	India	Expt. 1	AS	19	India
CC-P	7	70	20/200	India	Expt. 2	AJ	7	Germany
CC-Q	39	6	20/72	Germany	Expt. 2	AB	40	Germany
CC-Q	39	6	20/72	Germany	Expt. 1	BA	38	Germany
CC-R	46	24	20/373	Germany	Expt. 2	AC	46	Germany
CC-R	46	24	20/373	Germany	Expt. 1	AB	46	Germany
CC-S	45	24	20/20	Germany	Expt. 2	BC	39	Germany
CC-S	45	24	20/20	Germany	Expt. 1	AC	40	Germany
DC-A	18	24	20/30	India	Expt. 2	AX	19	India
DC-B	8	84	20/126	India	Expt. 1	AF	8	Germany

DC-C	11	120	20/30	India	Expt. 1	AE	10	Germany
DC-D	17	183	20/63	India	Expt. 2	AQ	16	India
DC-C	11	120	20/30	India	Expt. 1	AE	10	Germany
DC-E	9	85	20/25	India	Expt. 2	AG	9	Germany
DC-E	9	85	20/25	India	Expt. 1	AG	9	Germany
DC-F	17	30	20/25	India	Expt. 1	AR	18	India
DC-G	13	96	20/32	India	Expt. 2	AH	13	India
DC-G	13	96	20/32	India	Expt. 1	AV	13	India
DC-H	13	90	20/32	India	Expt. 2	AP	12	India
DC-H	14	90	20/32	India	Expt. 1	BD	16	India
DC-I	11	101	20/40	India	Expt. 2	AI	10	Germany
DC-I	11	101	20/40	India	Expt. 1	AP	12	India
DC-J	13	122	20/25	India	Expt. 2	AK	15	India
DC-J	13	122	20/25	India	Expt. 1	AM	15	India
DC-K	14	144	20/30	India	Expt. 2	AL	15	India
DC-K	14	144	20/30	India	Expt. 1	AY	14	India
DC-L	19	174	20/20	India	Expt. 2	AZ	19	India
DC-M	12	84	20/20	India	Expt. 1	AW	12	India

Notes: CC = congenital cataracts, DC = developmental cataracts; CF 1 m = able to count fingers at a distance of 1 m from the participant

Table S2. Results of the Statistical Analysis of Tactile Spatiotemporal Order Judgments in the congenital cataract (CC) group and a group of matched typically sighted (M-TS) participants.

Predictor	Effect and Confidence Interval	Test Statistic and *p*-value
Group	-0.000356 (-0.00169, 0.000984)	$\chi^2(1) = 0.27, p = 0.604$
Hand Position	0.00309 (0.00235, 0.00382)	$\chi^2(1) = 75.11, p < 0.001$
Experiment	-0.000309 (-0.00139, 0.000769)	$\chi^2(1) = 0.50, p = 0.479$
Group x Hand Position	-0.00198 (-0.00345, -0.000507)	$\chi^2(1) = 6.74, p = 0.009$
Group x Experiment	-0.000294 (-0.00245, 0.00186)	$\chi^2(1) = 0.07, p = 0.785$
Hand Position x Experiment	-0.00208 (-0.00355, -0.000614)	$\chi^2(1) = 8.08, p = 0.005$
Group x Hand Position x Experiment	0.00206 (-0.000881, 0.00499)	$\chi^2(1) = 1.97, p = 0.160$

Contrast	Simple Effect and Confidence Interval	Test Statistic and *p*-value
Expt. 1: Group x Hand Position	-0.003005 (-0.00516, -0.000849)	$t(54.3) = -2.79, p = 0.007$
Expt. 2: Group x Hand Position	-0.000948 (-0.00294, 0.001048)	$t(54.3) = -0.95, p = 0.346$
Expt. 1 & CC Group: Hand Position	0.000542 (-0.000982, 0.00207)	$t(54.3) = 0.71, p = 0.479$
Expt. 2 & CC Group: Hand Position	0.003547 (0.002022, 0.00507)	$t(54.3) = 4.67, p < 0.001$
Expt. 1 & M-TS Group: Hand Position	0.003653 (0.002242, 0.00506)	$t(54.3) = 5.20, p < 0.001$
Expt. 2 & M-TS Group: Hand Position	0.004601 (0.003190, 0.00601)	$t(54.3) = 6.54, p < 0.001$

Table S3. Results of the Statistical Analysis of Tactile Spatiotemporal Order Judgments in the developmental cataract (DC) group and a group of matched typically sighted (M-TS) participants.

Predictor	Effect and Confidence Interval	Test Statistic and *p*-value
Group	-0.000356 (-0.00169, 0.000984)	$\chi^2(1) = 0.65, p = 0.422$
Hand Position	0.00309 (0.00235, 0.00382)	$\chi^2(1) = 35.61, p < 0.001$
Experiment	-0.000309 (-0.00139, 0.000769)	$\chi^2(1) = 0.02, p = 0.885$
Group x Hand Position	-0.00198 (-0.00345, -0.000507)	$\chi^2(1) = 1.44, p = 0.230$
Group x Experiment	-0.000294 (-0.00245, 0.00186)	$\chi^2(1) = 1.09, p = 0.296$
Hand Position x Experiment	-0.00208 (-0.00355, -0.000614)	$\chi^2(1) = 0.00, p = 0.978$
Group x Hand Position x Experiment	0.00206 (-0.000881, 0.00499)	$\chi^2(1) = 0.45, p = 0.505$
**Contrast**	**Simple Effect and Confidence Interval**	**Test Statistic and *p*-value**
Expt. 1 & DC Group: Hand Position	0.000991 (0.000052, 0.00193)	$t(39.4) = 2.13, p = 0.039$
Expt. 2 & DC Group: Hand Position	0.001864 (0.000925, 0.00280)	$t(39.4) = 4.02, p < 0.001$

Expt. 1 & M-TS Group: Hand Position	0.001296 (0.000306, 0.00229)	$t(39.4) = 2.65, p = 0.011$
Expt. 2 & M-TS Group: Hand Position	0.001533 (0.000543, 0.00252)	$t(39.4) = 3.13, p = 0.003$

Notes: Simple effects of hand position were not warranted by an interaction but calculated as a sanity check, given the importance of the effect for our question.

Table S4. Results of the Statistical Analysis of Auditory Spatiotemporal Order Judgments in Experiment 2 in the congenital cataract group and a group of matched typically sighted participants.

Predictor	Effect and Confidence Interval	Test Statistic and *p*-value
Group	-0.00125 (-0.00376, 0.00126)	$\chi^2(1) = 1.05, p = 0.306$
Hand Position	-0.000404 (-0.00104, 0.000237)	$\chi^2(1) = 1.68, p = 0.195$
Group x Hand Position	-0.000819 (-0.0021, 0.000462)	$\chi^2(1) = 1.73, p = 0.189$

Table S5. Results of the Statistical Analysis of Auditory Spatiotemporal Order Judgments in Experiment 2 in the developmental cataract group and a group of matched typically sighted participants.

Predictor	Effect and Confidence Interval	Test Statistic and *p*-value
Group	-0.000114 (-0.00122, 0.000994)	$\chi^2(1) = 0.05, p = 0.827$
Hand Position	0.000123 (-0.000168, 0.000414)	$\chi^2(1) = 0.80, p = 0.371$
Group x Hand Position	0.0000652 (-0.000517, 0.000647)	$\chi^2(1) = 0.06, p = 0.812$

Table S6. Results of the Statistical Analysis of Tactile-Auditory Spatiotemporal Order Judgments in Experiment 2 in the congenital cataract group and a group of matched typically sighted participants.

Predictor	Effect and Confidence Interval	Test Statistic and *p*-value
Group	-0.0012 (-0.0036, 0.00119)	$\chi^2(1) = 1.07, p = 0.301$
Hand Position	0.00153 (0.00076, 0.0023)	$\chi^2(1) = 16.65, p < 0.001$
Group x Hand Position	-0.00107 (-0.00262, 0.000469)	$\chi^2(1) = 2.05, p = 0.152$

Table S7. Results of the Statistical Analysis of Tactile-Auditory Spatiotemporal Order Judgments in Experiment 2 in the developmental cataract group and a group of matched typically sighted participants.

Predictor	Effect and Confidence Interval	Test Statistic and *p*-value
Group	-0.000184 (-0.00161, 0.00125)	$\chi^2(1) = 0.07, p = 0.785$
Hand Position	0.000868 (0.000395, 0.00134)	$\chi^2(1) = 15.14, p < 0.001$
Group x Hand Position	-0.0000503 (-0.000996, 0.000895)	$\chi^2(1) = 0.01, p = 0.910$

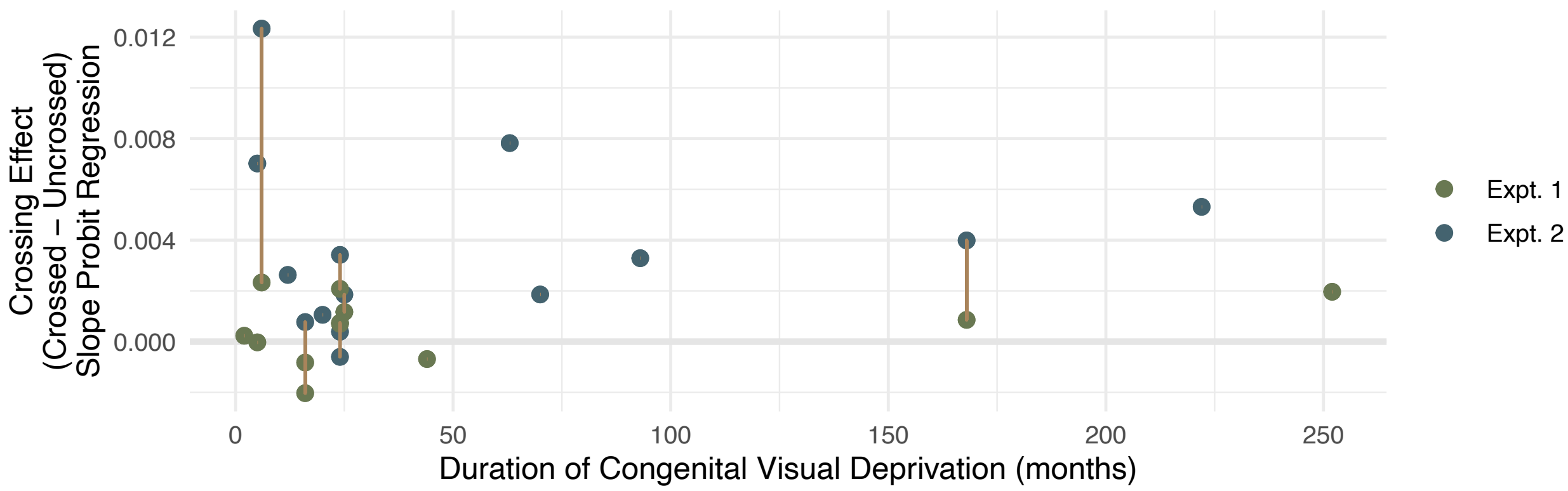

Figure S1. Crossing Effects and Duration of Congenital Visual Deprivation. Crossing effects in both experiments (Expt. 1 green, Expt. 2 blue) are shown as a function of the age at which the participant was treated for congenital cataracts. Brown vertical lines connect repeated measures by the same participant.
